# Does size‐selective harvesting erode adaptive potential to thermal stress?

**DOI:** 10.1002/ece3.11007

**Published:** 2024-02-07

**Authors:** Daniel E. Sadler, Stephan van Dijk, Juha Karjalainen, Phillip C. Watts, Silva Uusi‐Heikkilä

**Affiliations:** ^1^ Department of Biological and Environmental Science University of Jyväskylä Jyväskylä Finland

**Keywords:** adaptive potential, fisheries, phenotypic diversity, size selection, thermal stress

## Abstract

Overharvesting is a serious threat to many fish populations. High mortality and directional selection on body size can cause evolutionary change in exploited populations via selection for a specific phenotype and a potential reduction in phenotypic diversity. Whether the loss of phenotypic diversity that accompanies directional selection impairs response to environmental stress is not known. To address this question, we exposed three zebrafish selection lines to thermal stress. Two lines had experienced directional selection for (1) large and (2) small body size, and one was (3) subject to random removal of individuals with respect to body size (i.e. line with no directional selection). Selection lines were exposed to three temperatures (elevated, 34°C; ambient, 28°C; low, 22°C) to determine the response to an environmental stressor (thermal stress). We assessed differences among selection lines in their life history (growth and reproduction), physiological traits (metabolic rate and critical thermal max) and behaviour (activity and feeding behaviour) when reared at different temperatures. Lines experiencing directional selection (i.e. size selected) showed reduced growth rate and a shift in average phenotype in response to lower or elevated thermal stress compared with fish from the random‐selected line. Our data indicate that populations exposed to directional selection can have a more limited capacity to respond to thermal stress compared with fish that experience a comparable reduction in population size (but without directional selection). Future studies should aim to understand the impacts of environmental stressors on natural fish stocks.

## Graphical abstract

Size selection can act in tandem with diversity loss to reduce adaptive potential. Here, we show directional selection magnifies the effect of diversity loss alone to increase a population's susceptibility to thermal stress.

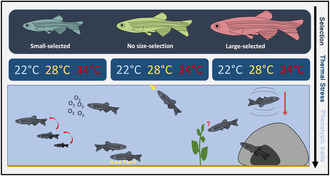

## 1 INTRODUCTION

Overharvesting can lead to rapid population declines (McCauley et al., 2015) causing a loss of genetic (Marty et al., 2015; Pinsky & Palumbi, 2014; Sadler et al., 2023; Therkildsen et al., 2019) and phenotypic variation (Olsen et al., 2009; Palumbi et al., 2019). A key aspect in many fisheries is the size selective removal of the largest individuals from the population (Jørgensen et al., 2007; Law, 2007; Lewin et al., 2006). Such directional selection on body size can drive evolutionary change towards specific phenotypic traits (Conover & Munch, 2002; Uusi‐Heikkilä et al., 2015), such as faster growth rate, earlier age at maturation and altered behaviour (Mollet et al., 2007; Olsen et al., 2004; Reid et al., 2023; Therkildsen et al., 2019; Uusi‐Heikkilä et al., 2015, 2017; van Wijk et al., 2013). A key question is whether altered phenotypic variation caused by directional selection and/or population decline decreases the resilience of species to changing environmental conditions (Morrongiello et al., 2021; Pörtner & Peck, 2010).

Population decline alone is likely to increase susceptibility to future stressors through random loss of adaptive alleles (Petrou et al., 2021; Thompson et al., 2019) and a reduction in phenotypic diversity (Anderson et al., 2008; Morrongiello et al., 2019). Many fisheries not only reduce population sizes substantially but also expose the targeted populations to size selection (i.e. directional selection), which can magnify the loss of genetic variation (Frankham, 2012), as favouring a specific phenotype can cause a concomitant directional shift in allele frequency (Quinn et al., 2007; but see Pinsky et al., 2021). While the effects of fisheries‐induced directional selection on phenotypic variation remain relatively understudied (but see Olsen et al., 2009; Palumbi et al., 2019), it is crucial to understand what consequences the additive effect of population decline and directional selection has on the average phenotype in a changing environment. Thus, not only do fish stocks have to cope with a loss of phenotypic diversity caused by reductions in population size but they may also be further limited by the reductions in phenotypic diversity resulting from directional selection (Groth et al., 2018; Marty et al., 2015; Therkildsen et al., 2019). It is unknown whether this loss of phenotypic diversity from directional selection is important, and how this could influence resilience to environmental stressors.

Exploited populations experience diverse stressors alongside fishery impacts (Planque et al., 2010; Wootton et al., 2021). An important stress experienced in many natural populations is temperature change (Mittelbach et al., 2007; Parmesan et al., 2003). Environmental temperature will likely rise beyond the tolerable limits of many fish species due to climate change and associated extreme weather events (Hollowed et al., 2013; Perry et al., 2010). Indeed, extreme weather events can lead to sea surface temperature increases of 2–4°C, and sometimes >5°C (Sen Gupta et al., 2020). Thermal stress can negatively affect fish populations through, for example, changes in life‐history traits including growth and reproduction (Pörtner & Farrell, 2008).

The temperature size rule (TSR) describes the growth response of ectotherms to temperature, with higher temperature predicted to select for fast growth and small adult body size (Atkinson, 1994; Cheung et al., 2013). Therefore, size‐selective harvesting and increased water temperature may interact and favour small body size (Audzijonyte et al., 2016). This possible combined effect of directional selection for body size and thermal stress may further negatively affect population growth and recruitment, and associated phenotypes (Planque et al., 2010; Rouyer et al., 2012; Wootton et al., 2021). It is important to understand how populations exposed to size‐selective fisheries cope with thermal stress and whether fisheries‐induced selection interacts with thermal stress to accelerate the effects of directional selection.

We studied experimentally how zebrafish (*Danio rerio*) that had experienced three size selection regimes responded to thermal stress. Size‐selection lines consisted of (1) small‐selected fish (a treatment that mimics the selection pattern typical to fisheries), (2) large‐selected fish (to understand the full range of responses caused by size selection) and (3) random‐selected fish (no size selection). We exposed fish from each selection line for 250 days to three temperatures—low (22°C), ambient (28°C) and elevated (34°C)—to determine if (1) directional selection impacted the response to thermal stress and whether (2) selection for a distinct (i.e. small or large) body size interacted with the response to a change in temperature. To provide a multivariate assessment of response to thermal stress, we monitored the growth, reproductive success, metabolic rate, behaviour and critical thermal maximum (CT_max_) of zebrafish. We hypothesised that (1) size‐selected fish (small‐ and large‐selected) would perform worse than random‐selected fish under thermal stress due to, for example, the potentially stronger loss of (genetic and phenotypic) diversity that occurs when there is directional selection compared with a reduction in population size, or, alternatively, that (2) size selection may interact with temperature, favouring small body size. The latter would lead us to hypothesise that small‐selected fish would perform better in high temperatures than large‐ and/or random‐selected fish following the TSR. We found that directional selection on body size (whether for large or small body size) magnified the negative consequences of thermal stress in terms of growth compared with a comparable population reduction but no directional selection (random‐selected fish). However, we found no evidence of an interaction between size selection and TSR.

## 2 MATERIALS AND METHODS

### 2.1 Study design

Selection lines were created using wild‐caught zebrafish (*Danio rerio*), from the West Bengal region of India (Uusi‐Heikkilä et al., 2010). In this experiment, we used fish from the three previously established selection lines (with two replicates each) that had been subject to 75% fishing mortality rate (Uusi‐Heikkilä et al., 2015): (1) small‐selected (where 25% of the smallest fish were kept in spawning stocks), (2) large‐selected (where 25% of the largest fish were kept in spawning stocks) and (3) random‐selected (where 25% randomly chosen fish were kept in spawning stocks). In each generation, all fish in a spawning stock (i.e. 25% of the population) were allowed to spawn (using multiple spawning boxes that each contained two females and four males). Harvesting continued for five generations, after which the selection lines phenotypically and genetically differed (Uusi‐Heikkilä et al., 2015, 2017). Next, the selection lines were allowed to recover (i.e. no harvesting) for 10 generations. We used these lines in the current experiment, as they exhibited persistent genetic differences (Uusi‐Heikkilä et al., 2017) and behavioural differences (Roy & Arlinghaus, 2022; Sbragaglia et al., 2019).

Prior to the thermal stress experiment, adult zebrafish were kept at 28°C with a 14:10 light cycle and fed ad libitum with a mixture of dry food (TetraMin XL) and live *Artemia salina*. During the experiment, fish were fed dry food ad libitum twice daily. Fish from each selection line replicate were exposed to three different temperature regimes at age 50 days post fertilisation (dpf): low (22°C), ambient (28°C) and elevated (34°C) for 250 days. To avoid high mortality at early (<50 dpf) life stages, we focused on later life stage effects. Ambient temperature is the control temperature, as it has been the standard rearing temperature in the laboratory for 15 generations as well as representing the natural environment of zebrafish (Sundin et al., 2019). Elevated and low temperatures were chosen as ±6°C from the ambient temperature, representing thermal stress on zebrafish physiology (Åsheim et al., 2020; Morgan et al., 2022) with +6°C also representing a potential temperature rise in extreme weather events (Sen Gupta et al., 2020).

Fish were tagged with visible implant tags (VIE; Northwest Marine Technologies, 162 Shaw Island, WA, USA) so that we could measure individual‐level data for all fish. We picked 360 individuals randomly from the selection line replicates for the experiment (*n* = 20 per selection line replicate, per temperature treatment). For each temperature treatment, we had three replicate 30 L glass tanks. In each tank, there were eight cylindrical wire mesh cages and, in each cage, we placed five zebrafish randomly distributed accorded to line (i.e. all individuals from the same line were not in the same tank, preventing confounding effects). Individuals were used as the unit of replication. Experimental fish were acclimated for 2 weeks at 28°C. Temperature was altered by ±1°C day^−1^ until it reached either 22°C or 34°C in the low and elevated temperature treatments, respectively.

### 2.2 Growth rate

Standard length (SL) and wet mass (WM) of 360 fish (*n* = 20 per selection line replicate per temperature treatment) were recorded weekly by placing individuals under anaesthesia (2‐phenoloxyethanol, 1.5% concentration). Fish were identified using a UV light on the VIE, and photographed (against millimetre paper for scale) using a Canon EOS 90D DSLR Camera affixed with a Sigma 105 mm DG Macro HSM lens. Images were measured using ImageJ (Schneider et al., 2012). WM was measured using an analytical balance (Mettler AE240). After approximately 100 days, the growth rate began to plateau and length/weight measurements were taken biweekly. Alongside weekly growth rate, specific growth rate was calculated for mass and length (ln final length (or weight) − ln initial length (or weight)/days × 100).

### 2.3 Reproductive success

Fish (*n* = 12 per selection line replicate per temperature treatment) were placed in pairs (one female, one male) in 1 L breeding boxes attached to 3.5 L tanks on a Techniplast housing rack and allowed to spawn for 7 days. Eggs were collected and counted for the number of fertilised, unfertilised and dead eggs. Fecundity was determined as an average number of fertilised eggs per breeding couple across the 7‐day spawning trial. We measured egg and egg yolk diameter of the fertilised eggs using a microscope (Olympus sz61 with a sc50 attachment) and then placed the eggs in a 24‐well plate incubated at the corresponding treatment temperature using the same tank water. Eggs were then checked once per day to monitor mortality rate and age at hatching (d). When larvae hatched, we measured the larval SL using the same microscope as with measuring the eggs.

### 2.4 Metabolic rate

We kept individuals (*n* = 10 per selection line replicate per temperature treatment) without food for 24 h before taking body mass and SL to allow calculation of mass‐specific metabolic rates. Oxygen consumption of four fish was measured per time interval individually in four replicate acrylic cylinders (volume 108 cm^3^) of the intermittent‐flow respirometer (Loligo® Systems, SY21020, Viborg, Denmark). The system was submerged in a heated water bath regulated with an E100 1.6 Kw heater, set according to treatment temperature, and placed in a climate regulated room in complete darkness (with fish unable to see each other during the experiment). Oxygen was measured using a fibre‐optic sensor on the OXY‐4 mini oxygen meter system and AutoResp software (Loligo Systems, Viborg, Denmark). Measurements were taken using an intermittent flow system every 25 min (including 220 s flush, 230 s delay and 1050 s closed measuring periods). Duration of measurement period for each set of four fish was 8 h. After each treatment, the system was cleaned with bleach and water replenished. Background bacterial oxygen consumption was measured before and after the experimental period by measuring the empty chamber without fish and subtracted from the oxygen consumption values of the fish. AutoResp calculated the decrease in oxygen consumption during closed periods, converted them automatically to respiration rates (mgO_2_h^−1^) per fish during each closed period and mass‐specific rates were obtained by dividing the respiration rates by fish mass (g WM). Standard metabolic rate (mass‐specific mgO_2_g^−1^ h^−1^; SMR) was calculated using the average of the three lowest respiration rates while maximum metabolic rate (mass‐specific mgO_2_^−1^gh^−1^; MMR) used the average of the three highest respiration rates. MMR in the intermittent respirometer was the handling stress induced maximum metabolic rate which has been observed to be near the maximum metabolic rate of fish in the swimming respirometer (Karjalainen et al., 1995). Absolute aerobic scope (mass‐specific mgO_2_g^−1^ h^−1^; AAS) was calculated as the difference between SMR and MMR to estimate the fish's ability to increase metabolic rate above maintenance level.

### 2.5 Behaviour

Exploration, boldness and feeding behaviour were measured (*n* = 10 per selection line replicate per temperature treatment). Fish were kept without food for 24 h before the trials. Behavioural trials occurred in a glass tank (30 L) divided into two sections by an opaque plastic sheet. One compartment was darkened and acted as a refuge, while the other compartment contained stones and a novel object (coloured tiles) and thus acted as an area for exploration (Figure S1). Fish were acclimated in the refuge area for 10 min before the divider was removed and then fish were allowed to explore the novel environment for 20 min. Exploration and boldness were measured as time exploring a novel environment (Le Roy et al., 2021) and the latency of emergence from a refuge (Krause et al., 1998), respectively. Feeding behaviour was recorded for 5 min by adding approximately 6 mg of flake food (TetraMin XL) and measuring the frequency fish took food from the water surface and the time taken to start feeding. After each trial, excess food was removed from the tank. Behaviour was filmed throughout the duration with an overhead view and a side view using a GoPro 7 Silver and a Canon EOS 90D. All trials were performed within an isolated, temperature‐controlled room to prevent disturbance. Behaviour videos were viewed at 1.5x speed to measure exploration time (s), number of emergences, latency of emergence (s), feeding frequency (number of feeding events), probability to feed and latency to feed (s).

### 2.6 Critical thermal maximum (CT_max_ )

Six fish per selection line replicate per temperature treatment were simultaneously placed in a 20 L thermal tank with a Lauda E100 1.6Kw heater separated with a mesh divider. The starting temperature corresponded to the experimental rearing temperature, and the water was heated at 0.3°C min^−1^ (Åsheim et al., 2020). Fish were observed until they experienced loss of equilibrium for 3 s (Becker & Genoway, 1979). Temperature of fainting (CT_max_) was then recorded, and the fish were immediately euthanized using an overdose of 2‐phenoloxyethanol. Thermal scope was calculated by subtracting the rearing temperature from the CT_max_.

### 2.7 Statistical analysis

#### 2.7.1 Univariate

All statistics were performed using R v.4.1.2 (R Core Team, 2022) within RStudio (Posit team, 2022). We used linear mixed models (LMMs) and generalised linear mixed models (GLMM) to analyse the effect of temperature treatment and selection line on life history (growth and reproduction), physiological (SMR, MMR, AAS and CT_max_) and behavioural traits (boldness, exploration and feeding behaviour) using temperature, selection line and their interaction as fixed effects. Individual fish were used as unit of replication in the growth rate analysis. Selection line replicate and rearing cage (and spawning tank for reproductive measures) were set as random effects (Trait ~ Temperature * Selection line + (1|cage) + (1|selection line replicate)). For growth (weight and length), we utilised log–log in the model to consider the non‐linearity of growth and used time as a fixed effect and the individual as a random effect (log(Size) ~ log(Week) * Temperature * Selection line + (Week|Cage) + (1|selection line replicate) + (1|individual)). If a model was in singularity and/or did not converge, we removed the corresponding random factor from the model preventing overfitting of the model after confirming no significant effect on the result. Analyses used *lmer* and *glmer* within the lme4 package (Bates et al., 2015) and the *lmertest* function within the lmerTest package (Kuznetsova et al., 2017). For data that did not fit the assumptions of the LMM, we used GLMM (details of the models in Tables S1 and S2). Post hoc pairwise comparisons of significant interactions were made using Tukey contrasts with *emmeans* function within the emmeans package (Length, 2023). Coefficients of variance (CVs) were calculated to assess differences in trait variability. CVs were bootstrapped (10,000 times) and compared using modified signed‐likelihood ratio tests within the cvequality package (Marwick & Krishnamoorthy, 2019).

#### 2.7.2 Multivariate

Permutational multivariate analysis of variance (PERMANOVA) was used to test for individual variation in multivariate phenotypic responses to treatments (temperature and selection line). Pairwise Gower distances were calculated using *vegdist* within the vegan package (Oksanen et al., 2013) to account for differences in scale between variables (Gower, 1971). The matrices produced were used in PERMANOVAs run (9999 permutations) using *adonis2*. Principal component analysis (PCA) was used to visualise multivariate phenotypes.

## 3 RESULTS

### 3.1 Growth rate

**FIGURE 1:**
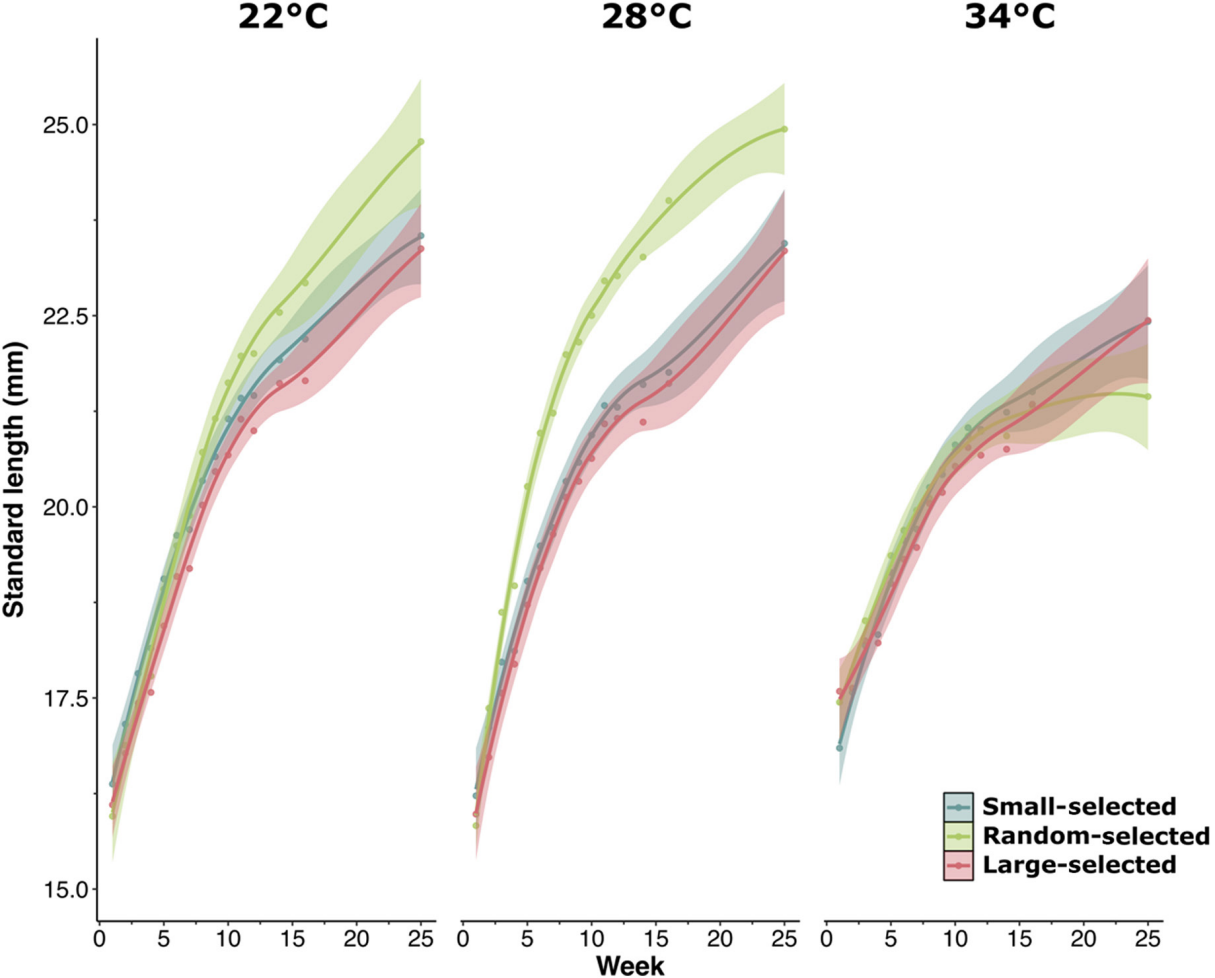
Differences in growth (standard length; mm) among zebrafish selection lines (large‐selected, random‐selected and small‐selected; selection‐line replicates combined) in the three temperature treatments: 22°C, 28°C and 34°C. Mean points and error around the lines represent standard error across housing cages within each treatment combination.

Weekly growth rate varied significantly among selection lines and temperatures in both SL (*F*_2,265_ = 4.80, *p* < .01, Table S1, Figure 1) and WM (*F*_2,263_ = 11.38, *p* < .001, Table S1, Figure S2). Specific growth rate and weekly growth rate were greater at 22°C and 28°C compared to the elevated 34°C temperature treatment (Figure 1, Figure S3, Table S1). Random‐selected fish had a faster growth rate than fish experiencing directional selection (small‐ and large‐selected fish) at 22°C and 28°C but not at 34°C, when all lines had comparable growth rates (Figure 1, Figure S3, Table S1). Both lines experiencing directional selection had similar growth rates across all temperatures (Figure 1, Figure S3, Table S1).

### 3.2 Reproductive success

**FIGURE 2:**
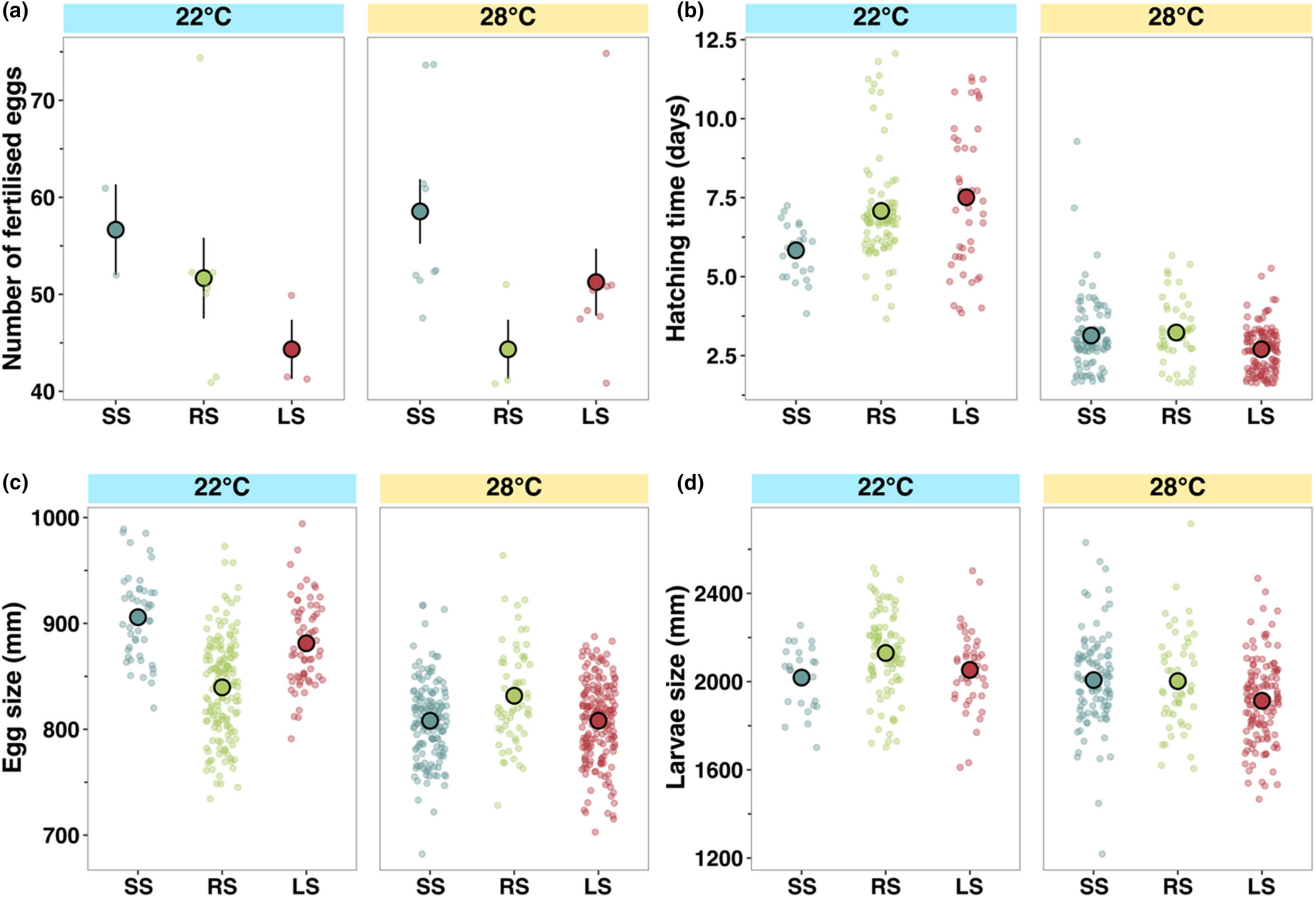
Differences in reproductive success among zebrafish selection lines (large‐selected (LS), random‐selected (RS) and small‐selected (SS); selection‐line replicates combined) in three temperature treatments. (a) The average number of fertilised eggs per female per spawning day, (b) larval age at hatch (days), (c) egg size (mm) and (d) larval size at hatch (mm). Data are shown as individual observations per fish (dots) and the mean with standard errors across housing cages within each treatment combination.

Fish housed at 34°C did not spawn regardless of the selection line. At 22°C and 28°C, fish did not significantly differ in fecundity among temperature treatments or selection lines (Figure 2a, Table S1).

Eggs produced by females exposed to the low temperature treatment (22°C) were significantly larger (*F*_1,666_ = 271.48, *p* < .001, Table S1, Figure 2b) than eggs produced at ambient temperature (28°C). Selection line also influenced egg size within both temperature treatments, with the random‐selected line having the smallest egg size at 22°C but the largest egg size at 28°C (*F*_2,666_ = 54.19, *p* < .001, Table S1, Figure 2b).

Larvae took a significantly longer time (*t* = 0.01, *p* < .001, Figure 2c, Table S2), by an average of 4 days, to hatch at 22°C than at ambient temperature (28°C). At 28°C, there were no differences in larval age at hatch among selection lines, but at 22°C, larvae from small‐selected lines hatched significantly earlier than larvae from random‐ and large‐selected lines (*t* = 0.015, *p* < .001, Figure 2c, Table S2).

Larvae hatched at 22°C were significantly larger than larvae hatched at 28°C (*F*_1,442_ = 19.57, *p* < .001, Figure 2d, Table S1). However, larval size at hatch did not significantly differ among selection lines (Table S1, Figure 2d).

### 3.3 Metabolic rate

**FIGURE 3:**
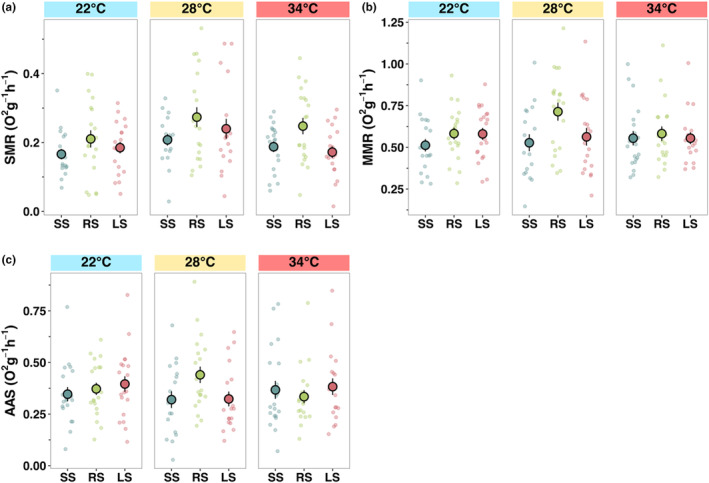
Differences in metabolic function among zebrafish selection lines (large‐selected (LS), random‐selected, (RS) and small‐selected (SS); selection‐line replicates combined) in three temperature treatments. (a) Mass‐specific standard metabolic rate (SMR), (b) maximum metabolic rate (MMR) and (c) absolute aerobic scope (AAS). Data are shown as individual observations per fish (dots) and the mean with standard errors across housing cages within each treatment combination.

There was no significant interaction between temperature and selection line in SMR, MMR or AAS. SMR was the highest at the ambient (28°C) temperature (*F*_2,166_ = 4.41, *p* < .05; Table S1, Figure 3a). Random‐selected lines showed a higher SMR than the lines experiencing directional selection: large‐selected (emmean contrast: −0.056, *p* < .05) and small‐selected (emmean contrast: 0.060, *p* < .01), corresponding with higher growth rate at low and ambient temperature (*F*_2,166_ = 5.36, *p* < .01; Table S1, Figure 3a). MMR differed between lines (*F*_2,166_ = 2.76, *p* < .05; Table S1, Figure 3b), with random‐selected having a significantly higher MMR than small‐selected (emmean contrast: −0.095, *p* < .05), but unlike SMR, it did not significantly differ across the three temperatures (Table S2, Figure 3b). AAS did not differ between lines or temperature (Table S2, Figure 3c).

### 3.4 Behaviour

**FIGURE 4:**
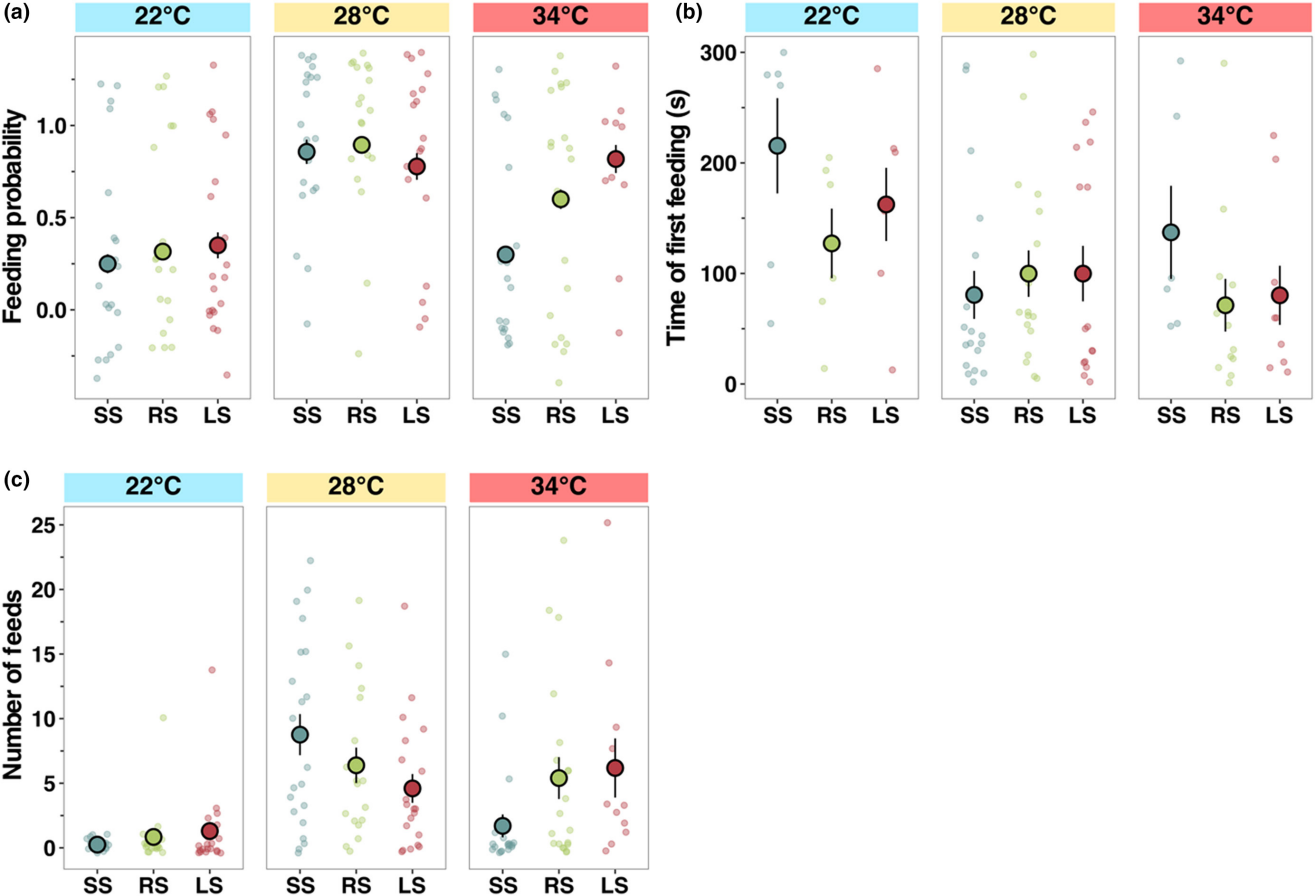
Differences in feeding behaviour among zebrafish selection lines (large‐selected (LS), random‐selected (RS) and small‐selected (SS); selection‐line replicates combined) in three temperature treatments. (a) Feeding probability, (b) time taken to reach first feeding attempt (s) and (c) number of feeding events within 5‐min feeding trial. Data are shown as individual observations per fish (dots) and the mean with standard errors across housing cages within each treatment combination.

Probability to feed differed among temperatures and selection lines, with feeding most likely to occur at ambient (28°C) and least likely at the low (22°C) temperature (*z* = −1.10, *p* < .05, Figure 4a, Table S2). Feeding latency differed significantly with temperature but not among selection lines (*F*_2,160_ = 1.56, *p* < .05, Figure 4b, Table S1), with fish at the lowest temperature taking longer to feed than at 28°C and 34°C (emmean contrast: 74.7, *p* < .05). Number of feeding events was significantly different between temperatures (*F*_2,160_ = 6.11, *p* < .05, Figure 4c, Table S1), with the highest being at ambient temperature and the lowest at 22°C regardless of the selection line (emmean contrast: −5.34, *p* < .001), since, at 22°C, many individuals did not feed (Figure 4c, Table S1).

**FIGURE 5:**
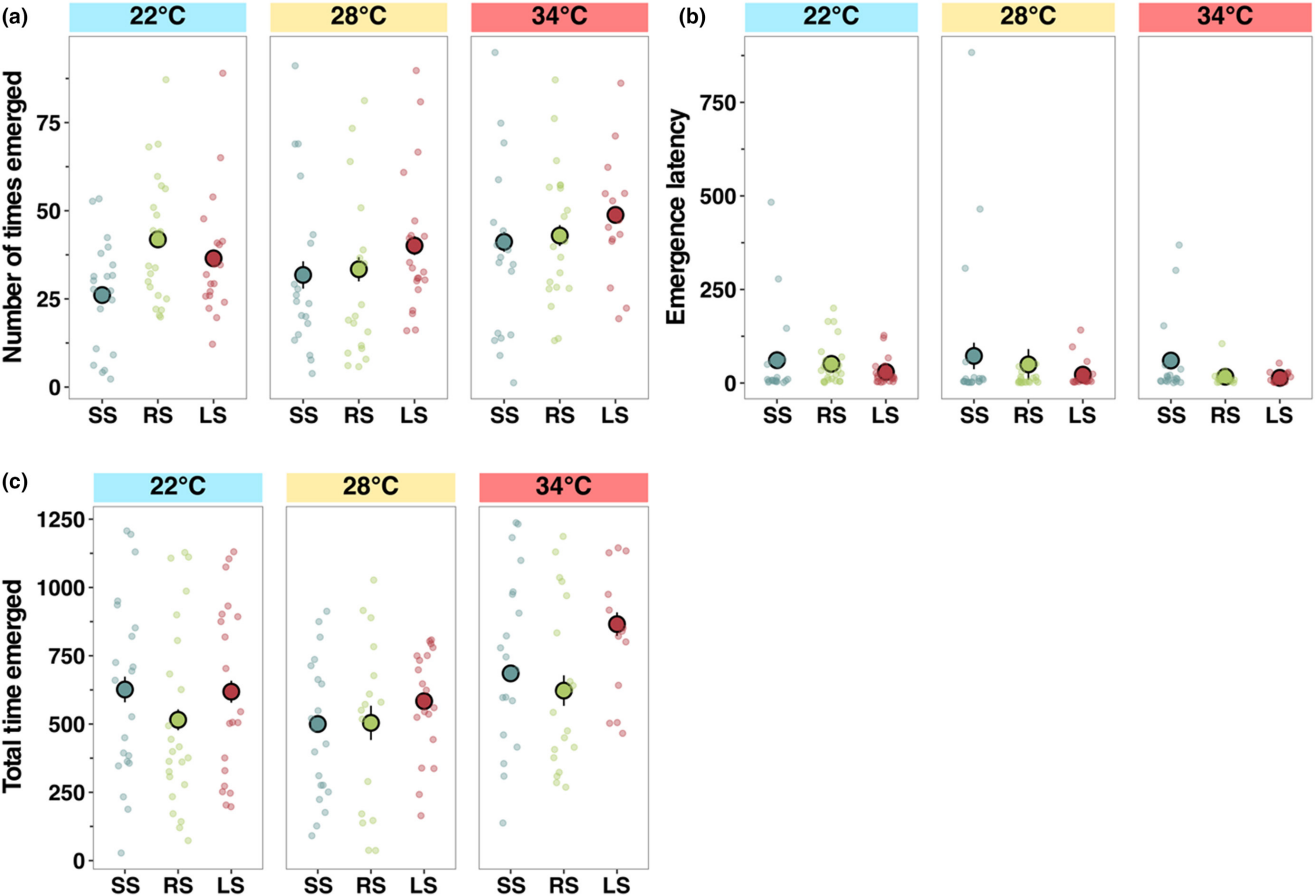
Differences in boldness and exploration among zebrafish selection lines (large‐selected (LS), random‐selected (RS) and small‐selected (SS); selection‐line replicates combined) in three temperature treatments. (a) Number of times emerged from the shelter within the 20‐min time period, (b) proxies of boldness measured as time taken to emerge from the shelter and (c) total time spent emerged from the shelter (an indicator of exploration). Data are shown as individual observations per fish (dots) and the mean with standard errors across housing cages within each treatment combination.

The number of times fish emerged from the shelter was most frequent at 34°C (*z* = 2.01, *p <* .05, Figure 5a, Table S2). Selection line had a significant effect as random‐selected fish emerged most frequently at 22°C (*z* = 2.46, *p* < .05, Figure 5a, Table S2). The latency time to emerge from the refuge (a proxy for boldness) was the highest at the low temperature (*z* = 2.39, *p* < .05, Figure 5b, Table S2), and the boldest individuals were from random and large‐selected lines, with the small‐selected lines taking the longest to emerge (*z* = 2.39, *p* < .05, Figure 5b, Table S2). Fish from the random‐selected and large‐selected lines were less explorative than fish from the small‐selected line at 28°C (RS; *z* = 4.80, *p* < .001, LS; *z* = 2.303, *p* < .05, Figure 5c, Table S2), but selection lines did not differ in explorative behaviour at the elevated or lower temperature (Figure 5c, Table S1).

### 3.5 Thermal tolerance

**FIGURE 6:**
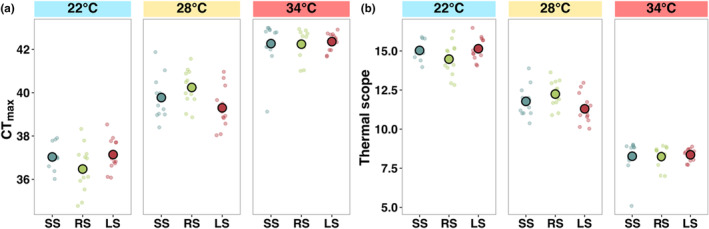
Differences in thermal limitations among selection lines of zebrafish (large‐selected (LS), random‐selected (RS) and small‐selected (SS); selection‐line replicates combined) in the three temperature treatments. (a) Critical temperature (CT_max_) where the fish loses equilibrium and (b) thermal scope (CT_max_ – acclimation temperature). Data are shown as individual observations per fish (dots) and the mean with standard errors across housing cages within each treatment combination.

CT_max_ followed a typical temperature‐dependent relationship, whereby CT_max_ increased with the rearing temperature (*F*_2,99_ = 359.40, *p* < .001; Figure 6a, Table S1). However, selection line had no significant effect on CT_max_ (Figure 6a, Table S1). Thermal scope (rearing temperature – CT_max_) decreased as temperature increased, suggesting fish at 34°C were close to their thermal limit (*F*_2,99_ = 534.60, *p* < .001; Figure 6b, Table S1). In contrast to CT_max_, there was a significant interaction between temperature treatment and selection line (*F*_2,99_ = 2.84, *p* < .05; Figure 6b, Table S1), whereby small‐selected fish had the lowest thermal scope at 28°C (emmean contrast: −5.41, *p* < .001) while the random‐selected line had the lowest thermal scope at 34°C (emmean contrast: −2.52, *p* < .001).

### 3.6 Mortality of experimental fish

Mortality increased as temperature increased, with the lowest number of mortalities at 22°C and the highest at 34°C (*z* = −2.723, *p* < .01, Table S2).

### 3.7 Multivariate phenotype

**FIGURE 7:**
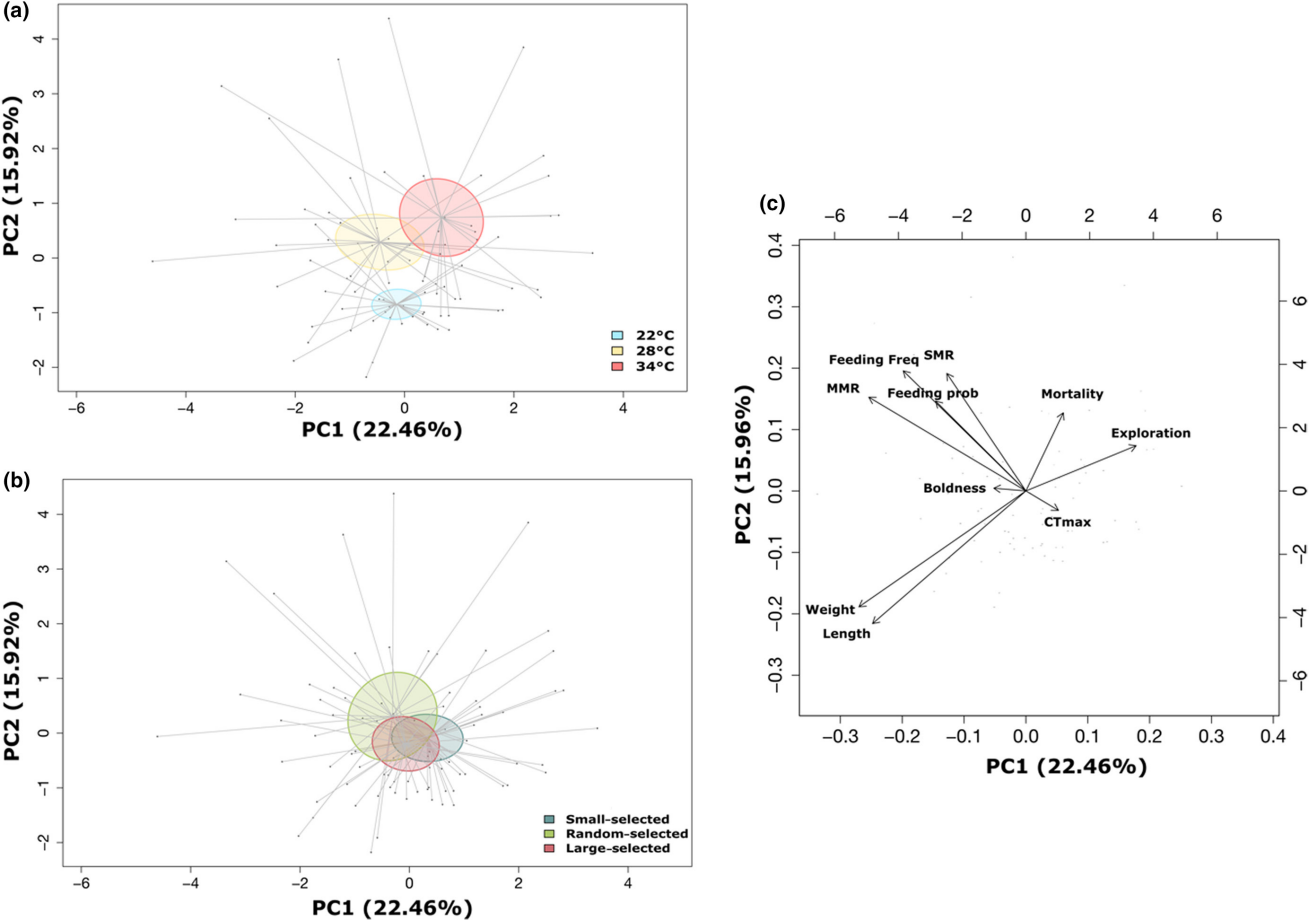
Principal component analysis of multiple measured traits with 95% confidence intervals across (a) zebrafish selection lines and (b) temperature treatments. Contributions to principal component space shown in biplot (c). Lengths of lines indicate distance of each individual from the respective group centroid.

Assessing coefficient of variation across univariate traits showed a small difference in variation across lines (Table S5); however, when all life‐history, physiological and behavioural traits were analysed together, temperature had a significant effect on the multivariate phenotype (PERMANOVA, *F*_1,77_ = 6.56, *p* < .001, Figure 7a, Table S3) but selection line did not (PERMANOVA, *F*_2,77_ = 1.79, *p* = .09, Figure 7b, Table S3). Despite no significant difference among the selection lines, there was greater variation in phenotypic responses to thermal stress in the random‐selected line as indicated by the larger ellipses (Figure 7b).

PC1 accounted for 22% of the phenotypic variation and mainly separated the mean phenotype at 34°C from the lower temperatures, while PC2 accounted for 16% of the variation, separating the mean phenotype at 22°C from the higher temperatures (Figure 7a). Individuals with higher PC1 scores were exposed to 34°C with higher mortality, greater exploration tendency and higher CT_max_ (Table S4). Individuals exposed to 28°C and 22°C exhibited faster growth (weight and length), were bolder, had higher metabolic rates (MMR and SMR), and greater feeding probability and frequency (Table S4). Individuals with high PC2 scores exposed to the 28°C and 34°C temperature treatments had higher mortality, metabolic rate, feeding probability and feeding frequency, boldness and exploration (Table S4, Figure 7c). While individuals at 22°C had a greater CT_max_ and growth rate (Table S4, Figure 7c). Moreover, when we subset PCAs by temperature, we show feeding rate is associated with growth rate at 28°C as expected under ambient (28°C) environmental conditions; however, this association weakens at the stressful temperatures (22°C and 34°C). Indeed, at 34°C, feeding rate becomes associated with metabolic rate instead (Figure S4).

## 4 DISCUSSION

Directional selection on a phenotypic trait can change the mean value and reduce diversity of the target trait and any correlated phenotypic traits, with the loss of diversity potentially affecting the ability of a population to respond to future stressors. Here, we show that directional selection on body size can exacerbate a population's susceptibility to thermal stress beyond that which would occur in a population that had experienced a random loss of diversity. In line with our first hypothesis, the random‐selected line exhibited a differential shift in their multivariate phenotype (Figure 7b; Pigliucci & Preston, 2004) and had greater fitness in terms of growth rate. Counter to our second hypothesis, we did not find evidence that fish selected for large or small body size would perform better at low or elevated temperature.

Variation in temperature drives large differences in phenotypes in many vertebrates, including fitness‐related traits such as growth (Atkinson, 1994; Killen et al., 2010), reproduction (Alix et al., 2020; Jonsson & Jonsson, 2019) and behaviour (Biro et al., 2010; Neubauer & Andersen, 2019). Metabolic rate is also a critical component of fitness (Killen et al., 2016) and is positively correlated with temperature in ectotherms (Clarke & Fraser, 2004; Morgan et al., 2022; Seebacher et al., 2014). However, beyond a critical temperature outside the thermal niche of an organism, metabolic rate starts to decline (Schulte, 2015; Schulte et al., 2011), as demonstrated in the present study. At the low temperature, metabolic rate was lower and the fish were less active than in the ambient temperature, potentially due to slowdown of biological processes (Volkoff & Rønnestad, 2020). Indeed, this slower pace of life is supported by longer developmental time (age at hatch), and larger larvae emergence at 22°C. Despite significant differences in metabolic rate (SMR and MMR), it is notable that our effect size was small, which could be evidence of metabolic acclimation occurring to the temperature during the long‐term experimental period (Sandblom et al., 2014). Indeed, acclimation in a stable environment allows some resilience to extreme temperature differences (Seebacher et al., 2014), and future studies should explore different acclimation temperatures in more detail. That exposure to an elevated temperature (34°C) prevented zebrafish from reproducing potentially reflects more energy being allocated to metabolic maintenance at this temperature (Donelson et al., 2010) and is consistent with studies that found teleost spawning being sensitive to increases of 2–3°C (Alix et al., 2020; Hotta et al., 2001). We show that long‐term exposure to elevated temperature had a significant negative effect on fish performance (growth, fecundity and survival) suggesting that their fitness may decrease under future extreme weather events.

Phenotypic traits covary genetically (Law, 1991) and phenotypically (Plaistow & Collin, 2014) and it is therefore important to assess multiple traits and understand their interconnectivity as a multivariate phenotype (Pigliucci & Preston, 2004), especially when a population undergoes selection, for example, fisheries selection. As such, although we show differing responses across the univariate phenotypic traits, studies on univariate traits may miss subtle but relevant phenotypic shifts (Plaistow & Collin, 2014). We show that covariation in phenotypic traits breakdown under stress, for example, growth rate was no longer correlated with food uptake at either lower or elevated thermal stress. The apparent breakdown in correlated traits in animals exposed to a stressful environment is comparable to a loss of correlation in copy number in genomes of mammals exposed to pollution (Jernfors et al., 2021). Here, we show that directional selection drives a shift in average phenotype (i.e. multivariate phenotype), leading to increased susceptibility to thermal stress, which in turn could alter phenotypic diversity (O'Dea et al., 2019).

Directional selection induces marked phenotypic changes in fish populations, such as reduced adult body size and earlier maturation at a smaller size (Conover & Munch, 2002; Uusi‐Heikkilä et al., 2015; van Wijk et al., 2013). Directional selection has a greater impact on phenotypic traits than a population reduction alone because it causes selective sweeps and can magnify loss of diversity via genetic hitchhiking (Frankham, 2012; Stephan, 2019; Therkildsen et al., 2019). It is speculated that such selection for specific phenotypes (e.g. body size) is associated with further loss in diversity (Frankham, 2012). Notably, the direction of directional selection (i.e. for small or large size) in our study had little effect as the small‐ and large‐selected lines had similar phenotypic responses to thermal stress in a number of traits. That both phenotypic outcomes of directional selection performed poorly suggests that some general mechanism might have reduced resilience to thermal stress, potentially related to inbreeding and further loss of diversity compared to random population reduction alone (Frankham, 2012). Our results therefore suggest the direction of selection per se may not matter and that size‐selective fisheries may erode phenotypic diversity further than non‐size‐selective fisheries.

The effect of size‐selective harvesting on fish populations is well studied (Pinsky & Palumbi, 2014; Therkildsen et al., 2019; van Wijk et al., 2013), but the interaction between reduced body size and warming is a relatively unexplored area in the fisheries context. At 34°C, random‐selected fish showed similar growth reductions to the size‐selected lines, suggesting this higher temperature had a severe effect upon all lines, regardless of prior selective pressures. Contrary to expectations following the TSR (Atkinson, 1994), both large‐ and small‐selected fish had similar phenotypic responses to suboptimal temperatures and performed equally in terms of growth and reproduction. While small‐selected fish were not more vulnerable to low or high temperatures compared with large‐selected fish in our study, in more stochastic, natural environments size truncation may decrease demographic buffering and population stability (Hočevar & Kuparinen, 2021; Kuparinen et al., 2016). Moreover, it is important to note that we used a large range in temperature (12°C) across the thermal scope of zebrafish (Morgan et al., 2022) that would likely be experienced by animals in nature for shorter infrequent timescales following extreme weather events (Sen Gupta et al., 2020). It would therefore be prudent for future studies to assess not only stable temperatures but also the impact of stochastic and variable temperature change that could disrupt possible acclimation.

Directional selection on body size can select for heritable behavioural traits that correlate with size, such as feeding behaviour, activity, exploration, boldness and aggression (Uusi‐Heikkilä et al., 2015; Walsh et al., 2006). Differences in feeding behaviour may be important, as food intake will directly impact growth rate, therefore fish that show reduced feeding probability could have lower growth rate. Here our patterns do not always match (e.g. fish reared at low temperatures fed less, but had the similar growth rate to those reared at 28°C) potentially due to laboratory conditions where fish are fed ad libitum and do not need to spend energy on avoiding predators and/or parasites. Behavioural response to thermal stress depended on the type of directional selection, that small‐selected fish were shyer than other lines is consistent with previous work (Monk et al., 2021; Sbragaglia et al., 2019; Uusi‐Heikkilä et al., 2015). Furthermore, fish were less bold in elevated temperatures. This might suggest that at least with certain fishing gear (e.g. angling), selection favouring small body size could lead to increased vulnerability to fishing and predation, and reduced foraging success (Alós et al., 2012, 2015; Diaz Pauli et al., 2015; Härkönen et al., 2014; Klefoth et al., 2012; Stamps, 2007). Overall, we did not detect as clear patterns in behavioural traits than in, for example, growth and physiology, which could be caused by the relatively high plasticity of behavioural traits compared to morphological and physiological traits (Duckworth, 2009; Mousseau & Roff, 1987). Our results suggest that behavioural responses to altered temperatures act differently than life history and physiological responses and are more dependent on the direction of selection.

In balanced harvesting, fishing mortality is not applied to selected functional groups, species or size of individuals and balanced harvesting has been suggested to reduce the negative effects of fisheries on ecosystems and on targeted populations by mitigating the effects of directional selection (Garcia et al., 2012). Balanced harvesting can increase stock productivity (Zhou et al., 2015), aid the recovery of populations' natural size structures (Beamish et al., 2006) and improve the resilience of the populations to natural disturbances (Hixon et al., 2014). Balanced harvesting, especially when it comes to body size, should maintain more phenotypic (and potentially genetic) variation in an exploited stock compared to size‐selective harvesting. In our experiment, the random‐selected line corresponds with balanced harvesting; however, this line did not maintain more phenotypic variation (with traits considered as a multivariate phenotype) than the size‐selected lines. However, random‐selected fish had greater fitness than small‐ and large‐selected fish, particularly when using adult body size and growth as a proxy for fitness (Barneche et al., 2018; White et al., 2013). Even if high fishing mortality without directional selection reduces phenotypic diversity through the reduction in population size, it should reduce less diversity than when combined with directional selection.

We show that random‐selected fish (no directional selection for body size) had the highest phenotypic variability in response to thermal stress. Size‐selective harvesting has a greater impact than the effects of a population reduction alone, with directional selection potentially magnifying loss of phenotypic diversity through selective sweeps and hitchhiking on covarying traits. Importantly, the direction of phenotypic change (either small‐ or large‐selected phenotype) appears less important than the action of directional selection per se, as size‐selective harvesting generally decreased the performance of fish exposed to thermal stress. A crucial next step would be to determine whether natural populations of exploited fish experience similar phenotypic changes under changing water temperature, and what are the underlying genetic mechanisms driving such changes. Our data suggest that selection regimes during harvesting should be reconsidered by utilising alternative harvesting strategies, aiming to reduce the magnifying effects of directional selection on fitness.

## AUTHOR CONTRIBUTIONS

**Daniel E. Sadler:** Conceptualization (equal); data curation (lead); formal analysis (lead); investigation (lead); methodology (equal); visualization (lead); writing – original draft (lead); writing – review and editing (equal). **Stephan van Dijk:** Conceptualization (equal); data curation (supporting); methodology (equal); writing – review and editing (equal). **Juha Karjalainen:** Methodology (equal); resources (equal); writing – review and editing (equal). **Phillip C. Watts:** Conceptualization (equal); investigation (equal); project administration (equal); resources (equal); supervision (equal); writing – review and editing (equal). **Silva Uusi‐Heikkilä:** Conceptualization (equal); funding acquisition (lead); investigation (equal); methodology (equal); project administration (lead); resources (equal); supervision (equal); writing – review and editing (equal).

## FUNDING INFORMATION

This work was supported by funding from the Academy of Finland (Grant no. 325107 (SUH)).

## CONFLICT OF INTEREST STATEMENT

No conflict of interest.

## Supporting information


Appendix S1

## Data Availability

All data are available in Dryad https://doi.org/10.5061/dryad.cvdncjt9v.
