# Supplementary material for: Does size‐selective harvesting erode adaptive potential to thermal stress?
Source: Ecol Evol. 2024 Feb 7;14(2):e11007. doi: 10.1002/ece3.11007 (PMC10850808; doi:10.1002/ece3.11007)
Supplement: Supplementary file 1 — Appendix S1 [file ECE3-14-e11007-s001.docx]

**Supporting information for**

**“****Does size-selective harvesting erode adaptive potential to thermal stress?”**

Authors: Daniel E. Sadler, Stephan van Dijk, Juha Karjalainen, Phillip C. Watts, Silva Uusi-Heikkilä

**Page Contents**

S2 Figure S1: Behaviour experiment design

S3 Figure S2: Growth curve for weight measurements

S4 Figure S3: Growth rate for weight and length

S5 Figure S4: Multivariate correlates per temperature

S6-S7 Table S1: LMM results

S7-S8 Table S2: GLMM results

S8 Table S3: PERMANOVA results

S9-S10 Table S4: Loading factors of parameters in Principal Component Analysis

S11-S13 Table S5: Coefficient of variance table

**
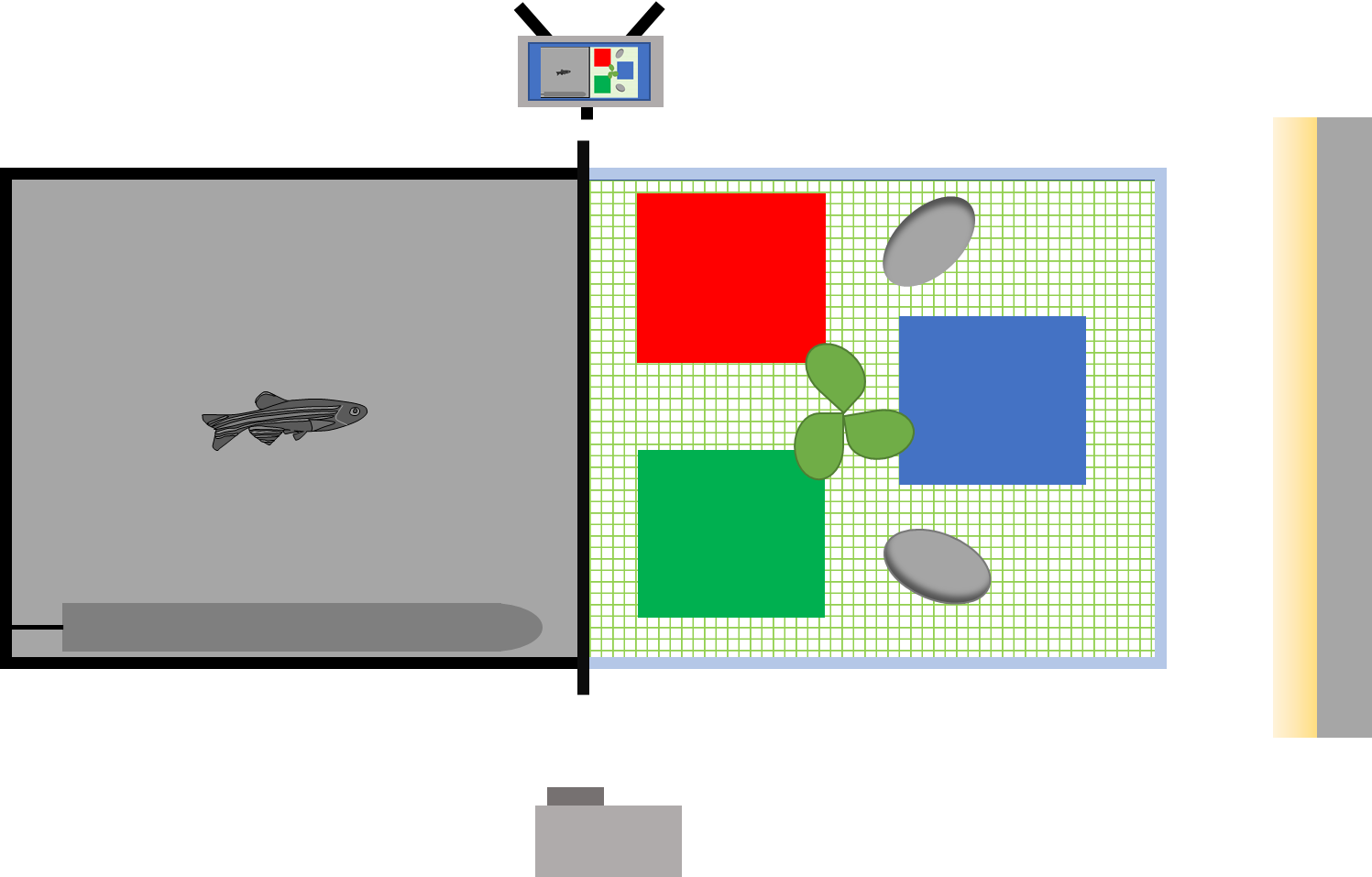
**

**C**

**B**

**B**

**B**

**A**

**A**

**Figure S1**: Top down view of the set up of the experimental tank for the behavioural experiment. Cameras were positioned above and to the side. Grey side represents sheltered side absent of light. Novel environment included (A) rocks, (B) coloured tiles and (C) plastic aquarium plant.


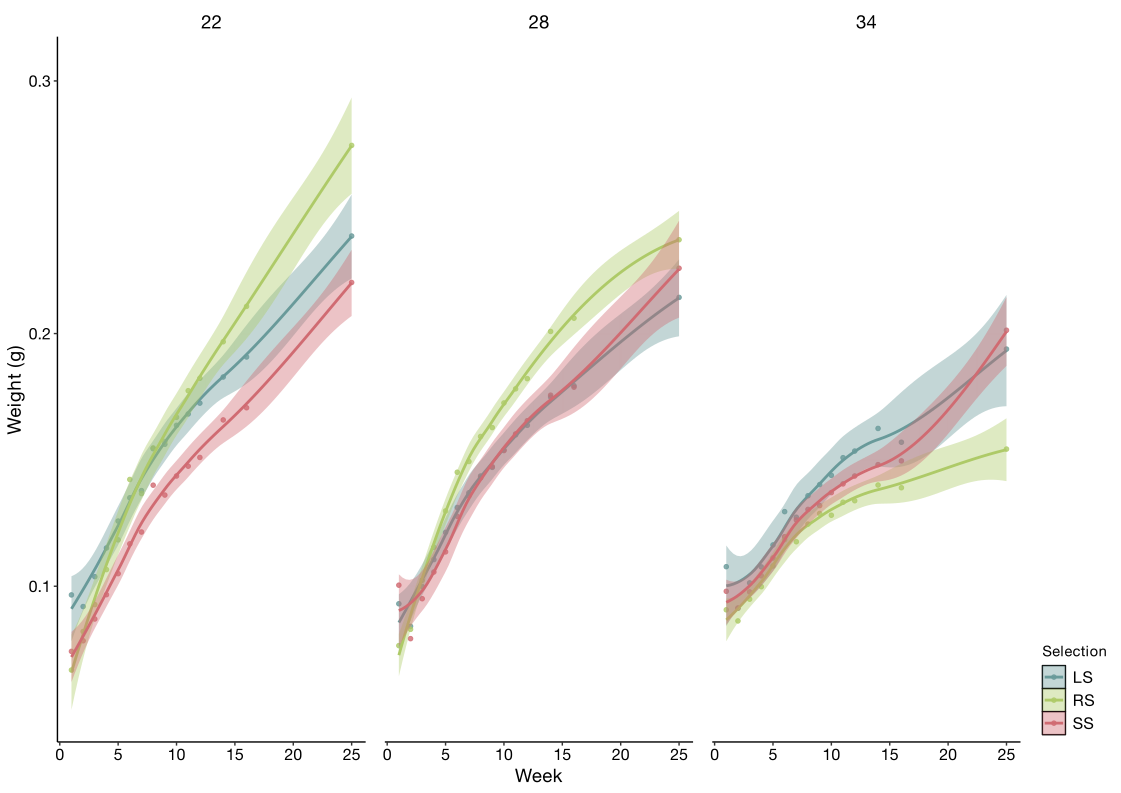


**Figure S2**: Differences in growth (weight; g) among the selection lines (large-selected, random-selected, and small-selected; selection-line replicates combined) in the three temperature treatments: 22°C, 28°C, and 34°C. Mean points and error around the lines represent standard error across housing cages within each treatment combination.


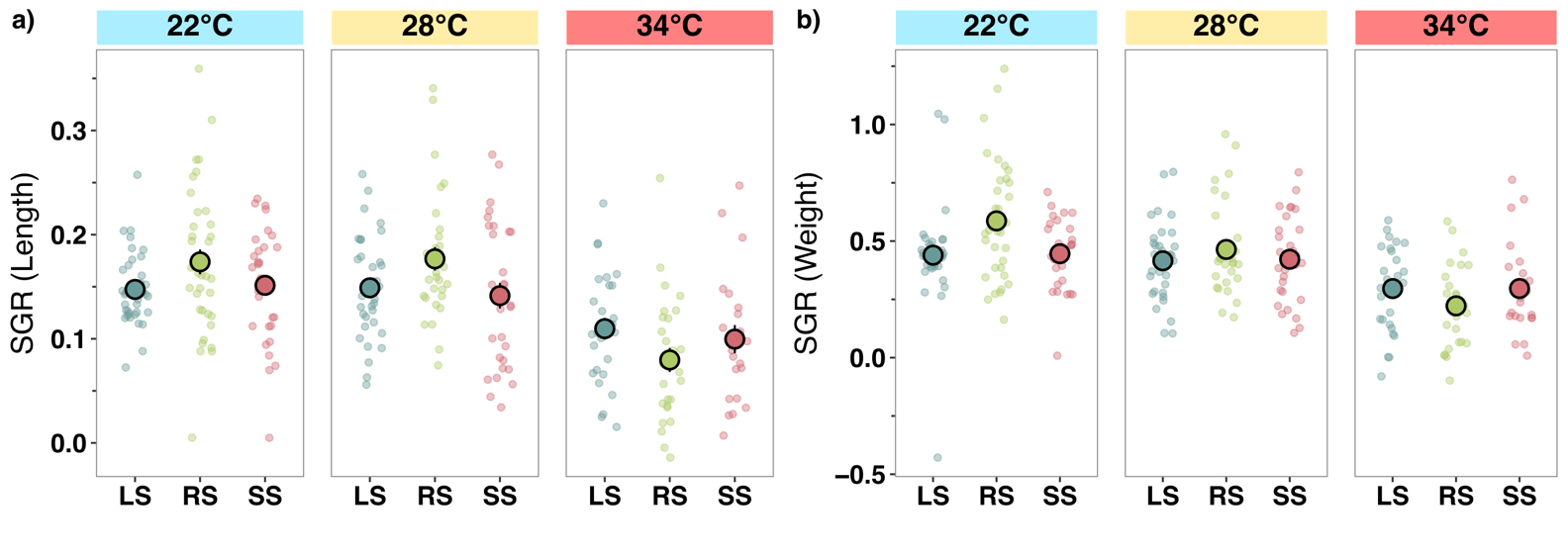


**Figure S3:** Variation in specific growth rate (SGR) (a) standard length (mm), (b) weight (g) among the selection lines: large-selected (LS), random-selected (RS), and small-selected (SS) in the three temperature treatments 22°C, 28°C, and 34°C. Mean points and error around the lines represent standard error across housing cages within each treatment combination.


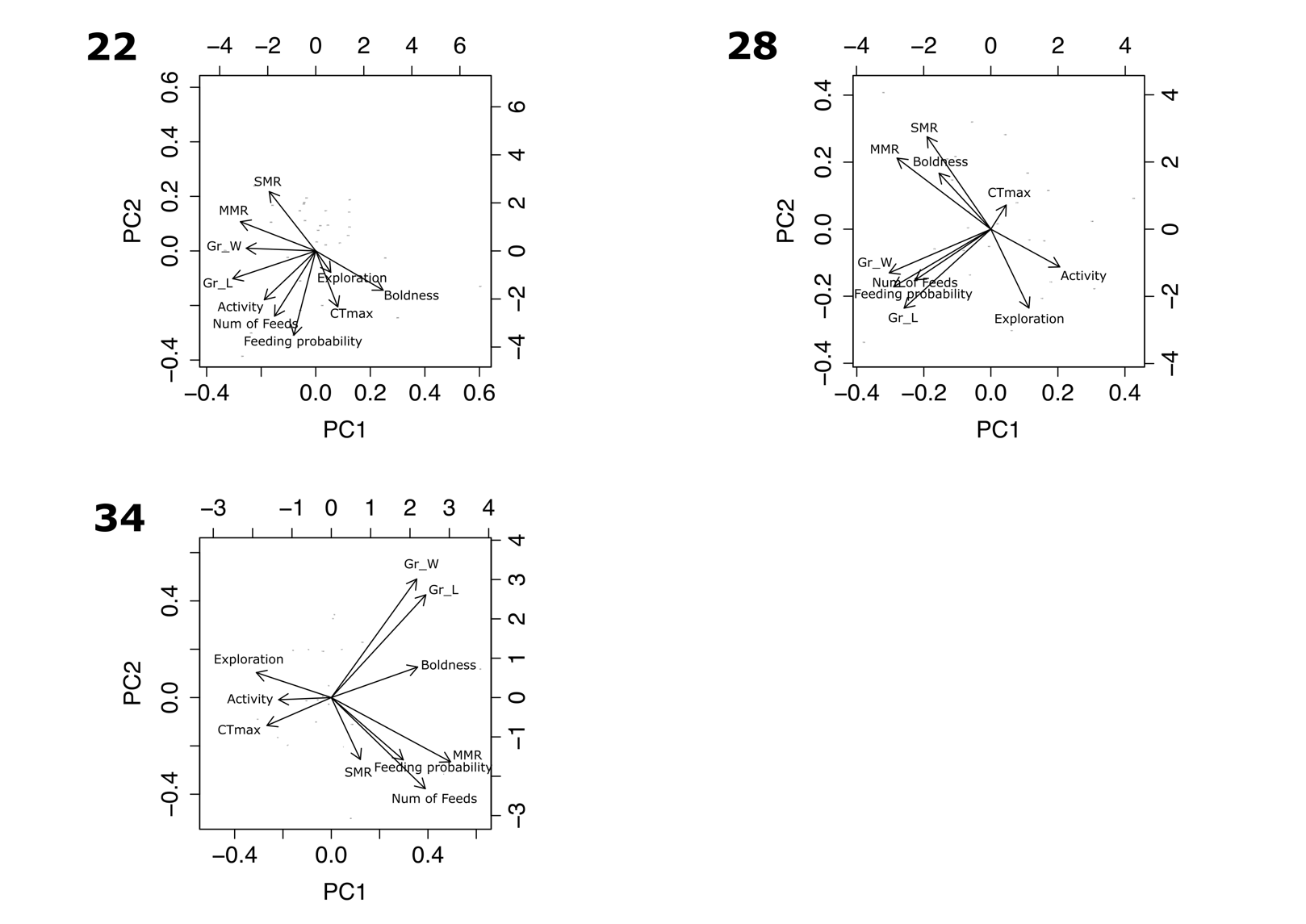


**Figure S4:** Principal component analysis of multiple measured traits. Contributions to prinicipal component space shown as biplots for 22°C, 28°C, and 34°C.

**Table S1:** Summary of measured traits analysed by LMMs

lmer(X~Selection*Temp +(1|Cage/Rep_Line) and lmer(X~Selection*Temp +(1|Cage/Rep_Line/mother) for reproductive traits. *If a random factor has no significant effect when imputed into the model, it is removed to prevent singularity and model convergence.*

| Trait | Treatment | Df | f value | *p value* |
| --- | --- | --- | --- | --- |
| Length  *log(Length) ~ log(Week) * Temperature * Selection + (Week \| Cage) + (1\|Line) + (1\|Rep_ID)* | log(Week) | 1 | 302.8156 | **<0.001 ***** |
|  | Temp | 1 | 9.8959 | **0.0022 **** |
|  | Selection | 2 | 1.6065 | 0.2060 |
|  | log(Week):Temp | 1 | 57.1119 | **<0.001 ***** |
|  | log(Week):Selection | 2 | 5.9859 | **0.0026**** |
|  | Temp:Selection | 2 | 1.6395 | 0.1994 |
|  | log(Week):Temp:  Selection | 2 | 4.7955 | **0.0085**** |
| Weight  *log(Weight) ~ log(Week) * Temperature * Selection + (Week \| Cage) + (1\|Line) + (1\|Rep_ID)* | log(Week) | 1 | 330.9379 | **<0.001 ***** |
|  | Temp | 1 | 12.6183 | **<0.001 ***** |
|  | Selection | 2 | 3.4461 | **0.0357*** |
|  | log(Week):Temp | 1 | 99.9635 | **<0.001 ***** |
|  | log(Week):Selection | 2 | 15.9350 | **<0.001 ***** |
|  | Temp:Selection | 2 | 2.7615 | 0.0677 |
|  | log(Week):Temp:  Selection | 2 | 11.3802 | **<0.001 ***** |
| Length (SGR) | Selection | 2 | 1.5981 | 0.2109 |
|  | Temp | 2 | 24.6091 | **<0.001 ***** |
|  | Selection: Temp | 4 | 2.5859 | **0.0463 *** |
| Weight (SGR) | Selection | 2 | 6.6572 | **0.0015**** |
|  | Temp | 2 | 24.9650 | **<0.001***** |
|  | Selection: Temp | 4 | 5.5085 | **0.0045**** |
| Fecundity | Selection | 2 | 1.3403 | 0.2793 |
|  | Temp | 1 | 2.0143 | 0.1677 |
|  | Selection: Temp | 2 | 1.3343 | 0.2808 |
| Egg Size | Selection | 2 | 59.401 | **<0.001***** |
|  | Temp | 1 | 271.479 | **<0.001***** |
|  | Selection: Temp | 2 | 54.193 | **<0.001***** |
| Larvae Size | Selection | 2 | 2.6914 | 0.5281 |
|  | Temp | 1 | 19.5738 | **0.0324*** |
|  | Selection: Temp | 2 | 3.2265 | 0.2972 |
| MMR | Selection | 2 | 2.7604 | **0.0339*** |
|  | Temp | 2 | 0.6751 | 0.51388 |
|  | Selection: Temp | 4 | 1.1326 | 0.35249 |
| SMR | Selection | 2 | 5.3621 | **0.0076**** |
|  | Temp | 2 | 4.4080 | **0.0169*** |
|  | Selection: Temp | 4 | 0.4917 | 0.7418 |
| Feeding Number | Selection | 2 | 0.4428 | 0.6447 |
|  | Temp | 2 | 6.1104 | **0.0169*** |
|  | Selection: Temp | 4 | 0.5353 | 0.5888 |
| Feeding Latency | Selection | 2 | 4.8663 | 0.2218 |
|  | Temp | 2 | 1.5558 | **0.0127*** |
|  | Selection: Temp | 4 | 1.0754 | 0.3812 |
| CT_max_ | Selection | 2 | 0.1040 | 0.9013 |
|  | Temp | 2 | 359.3990 | **<0.001***** |
|  | Selection: Temp | 4 | 2.8417 | **0.0281*** |
| Thermal Scope | Selection | 2 | 0.1040 | 0.9013 |
|  | Temp | 2 | 534.5985 | **<0.001***** |
|  | Selection: Temp | 4 | 2.8417 | **0.0281*** |

**Table S2:** Summary of measured traits analysed by GLMMs

| ***Trait*** | ***Treatment*** | ***Estimate*** | ***t/z value*** | ***p value*** |
| --- | --- | --- | --- | --- |
| AAS  +(1\|Cage) +(1\|LineRep)  *Family=Gamma* | (Intercept) | 2.8870 | 9.634 | **<0.001***** |
|  | Temp28 | 0.2388 | 0.533 | 0.595 |
|  | Temp34 | -0.1641 | -0.403 | 0.687 |
|  | SelRS | -0.1999 | -0.494 | 0.622 |
|  | SelLS | -0.3594 | -0.912 | 0.363 |
|  | Temp28:SelRS | -0.6541 | -1.143 | 0.255 |
|  | Temp34:SelRS | 0.4672 | 0.812 | 0.418 |
|  | Temp28:SelLS | 0.3280 | 0.540 | 0.590 |
|  | Temp34:SelLS | 0.2485 | 0.450 | 0.653 |
| Time emerged  (Exploration)  +(1\|Cage) +(1\|LineRep)  *Family=Gamma* | (Intercept) | 6.33849 | 35.254 | **<0.001***** |
|  | Temp28 | -0.76269 | -3.442 | **<0.001***** |
|  | Temp34 | 0.27863 | 1.096 | 0.273 |
|  | SelRS | -0.08375 | -0.342 | 0.732 |
|  | SelLS | 0.06033 | 0.246 | 0.806 |
|  | Temp28:SelRS | 1.19545 | 4.800 | **<0.001***** |
|  | Temp34:SelRS | 0.04669 | 0.142 | 0.887 |
|  | Temp28:SelLS | 0.74458 | 2.303 | **0.021*** |
|  | Temp34:SelLS | -0.27350 | -0.828 | 0.407 |
| Hatching Time  +(1\|Cage) +(1\|LineRep)  *Family=Gamma* | Intercept | 1.629311 | 0.31942 | **<0.001***** |
|  | SelectionRS | 0.037007 | 0.422587 | 0.930 |
|  | SelectionLS | -1.898011 | 0.398443 | **<0.001***** |
|  | Temperature | 0.133502 | 0.012059 | **<0.001***** |
|  | SelectionRS:  Temperature | -0.003541 | 0.016908 | 0.834 |
|  | SelectionLS:  Temperature | 0.07722 | 0.015583 | **<0.001***** |
| Feeding probability  *Family=binomial* | (Intercept) | -1.09861 | -2.127 | **0.033382** |
|  | Temp28 | 2.89037 | 3.57 | **0.000357** |
|  | Temp34 | 0.25131 | 0.354 | 0.72354 |
|  | SelRS | 0.32542 | 0.456 | 0.6487 |
|  | SelLS | 0.47957 | 0.688 | 0.491703 |
|  | Temp28:SelRS | 0.02288 | 0.019 | 0.984879 |
|  | Temp34:SelRS | 0.92734 | 0.948 | 0.343078 |
|  | Temp28:SelLS | -0.94958 | -0.87 | 0.38444 |
|  | Temp34:SelLS | 1.8718 | 1.62 | 0.105314 |
| Emergence Latency (Boldness)  *+(1\|Cage),*  *Family=Gamma* | Intercept | 0.022459 | 2.389 | **0.0169*** |
|  | Temp28 | 0.023581 | 2.004 | **0.045*** |
|  | Temp34 | 0.023934 | 2.028 | **0.0426*** |
|  | TreatRS | 0.021222 | 2.059 | **0.0395*** |
|  | TreatLS | 0.01935 | 1.719 | 0.0857 |
| No. of Emergences (Activity)  *+(1\|Cage) +(1\|LineRep)*  *Negativebinomial* | (Intercept) | 3.2541 | 22.988 | **<0.001***** |
|  | Temp28 | 0.1985 | 1.101 | 0.271 |
|  | Temp34 | 0.3741 | 2.005 | **0.0449*** |
|  | SelRS | 0.4816 | 2.459 | **0.0139*** |
|  | SelLS | 0.3288 | 1.629 | 0.1034 |
|  | Temp28:SelRS | -0.5849 | -2.269 | **0.0232*** |
|  | Temp34:SelRS | -0.3647 | -1.417 | 0.1566 |
|  | Temp28:SelLS | -0.1054 | -0.415 | 0.6781 |
|  | Temp34:SelLS | -0.1063 | -0.389 | 0.697 |
| Mortality  *GLM*  *Family=*  *binomial* | (Intercept) | 5.68185 | 3.486 | **<0.001***** |
|  | SelectionRS | -0.61636 | -0.278 | 0.78076 |
|  | SelectionLS | -2.67698 | -1.307 | 0.19118 |
|  | Temperature | -0.14602 | -2.723 | **0.00647**** |
|  | SelectionRS:T | 0.01511 | 0.206 | 0.83656 |
|  | SelectionLS:T | 0.07284 | 1.064 | 0.28727 |

**Table S3:** Summary of PERMANOVA results

| ***Treatment*** | ***Df*** | ***F Value*** | ***p value*** |
| --- | --- | --- | --- |
| *Selection* | 2 | 1.7893 | 0.0916 |
| *Temp* | 1 | 6.5593 | **<0.001***** |
| *Selection*Temp* | 2 | 1.5080 | 0.1617 |
| *Residuals* | 77 |  |  |

**Table S4**: Loading factors of life history parameters in Principal Component Analysis on individuals with selection lines and temperature treatments

|  | PC1 | PC2 | PC3 | PC4 | PC5 | PC6 | PC7 |
| --- | --- | --- | --- | --- | --- | --- | --- |
| Standard deviation | 1.4986 | 1.2617 | 1.225 | 1.0453 | 0.99511 | 0.92965 | 0.8543 |
| Proportion of variance | 0.2246 | 0.1592 | 0.1501 | 0.1093 | 0.09902 | 0.08643 | 0.07298 |
| Cumulative Proportion | 0.2246 | 0.3838 | 0.5339 | 0.6431 | 0.74215 | 0.82857 | 0.90155 |
|  |  |  |  |  |  |  |  |
|  | PC1 | PC2 | PC3 | PC4 | PC5 | PC6 | PC7 |
| Mortality | 0.1075 | 0.268549 | -0.38316 | 0.146322 | -0.13897 | 0.706658 | -0.47197 |
| Weight_Gr | -0.47956 | -0.39821 | -0.11418 | 0.162485 | 0.064987 | 0.189596 | 0.072492 |
| Len_Gr | -0.44052 | -0.45585 | -0.01034 | 0.169733 | 0.100139 | 0.272573 | 0.045037 |
| SMR..g.hr. | -0.22713 | 0.403793 | -0.51351 | -0.05948 | 0.189348 | -0.1822 | 0.135678 |
| MMR..g.hr. | -0.45132 | 0.322189 | -0.29229 | -0.01833 | -0.0497 | -0.10462 | 0.288151 |
| Num_feed | -0.35244 | 0.412746 | 0.39538 | 0.03118 | 0.095154 | 0.078004 | -0.07736 |
| Fed | -0.25947 | 0.309101 | 0.553599 | 0.103209 | 0.12578 | 0.110052 | -0.1895 |
| Time.emerged | -0.09228 | 0.009343 | 0.125904 | -0.72434 | -0.4096 | 0.385977 | 0.330942 |
| Total.time.emerged | 0.316725 | 0.155556 | 0.101381 | 0.409092 | 0.192545 | 0.382636 | 0.715829 |
| Temperature | 0.093553 | -0.06686 | -0.04462 | -0.46461 | 0.835701 | 0.180735 | -0.06851 |

|  | PC8 | PC9 | PC10 |
| --- | --- | --- | --- |
| Standard deviation | 0.62199 | 0.58679 | 0.50325 |
| Proportion of variance | 0.03869 | 0.03443 | 0.02533 |
| Cumulative Proportion | 0.94024 | 0.97467 | 1 |
|  |  |  |  |
|  | PC8 | PC9 | PC10 |
| Mortality | 0.025202 | -0.07226 | -0.02858 |
| Weight_Gr | -0.23452 | 0.177429 | -0.66341 |
| Len_Gr | 0.16824 | 0.01999 | 0.673919 |
| SMR..g.hr. | -0.29547 | 0.540653 | 0.225977 |
| MMR..g.hr. | 0.140634 | -0.69771 | -0.06092 |
| Num_feed | 0.621071 | 0.340741 | -0.15865 |
| Fed | -0.63969 | -0.1686 | 0.136132 |
| Time.emerged | -0.101 | 0.114882 | 0.034802 |
| Total.time.emerged | -0.02076 | 0.035516 | -0.0163 |
| Temperature | 0.05842 | -0.1575 | -0.07035 |

| Trait  **Table S5:** Bootstrapped Coefficient of variance values (n=10,000) per trait, differences assessed through Modified signed-likelihood ratio test. | Temperature | Line | Bootstrapped CV | MSLRT | P value |
| --- | --- | --- | --- | --- | --- |
| Length | 22 | LS | 35.99±5.85 | 8.774 | **0.012*** |
|  |  | RS | 41.10±5.38 |  |  |
|  |  | SS | 23.71±3.30 |  |  |
|  | 28 | LS | 48.23±5.14 | 3.701 | 0.151 |
|  |  | RS | 35.07±4.77 |  |  |
|  |  | SS | 33.64±3.76 |  |  |
|  | 34 | LS | 63.61±9.06 | 3.617 | 0.27 |
|  |  | RS | 75.35±11.78 |  |  |
|  |  | SS | 49.27±6.78 |  |  |
| Weight | 22 | LS | 32.86±6.04 | 3.711 | 0.158 |
|  |  | RS | 43.07±4.77 |  |  |
|  |  | SS | 48.50±15.23 |  |  |
|  | 28 | LS | 42.99±5.08 | 0.110 | 0.959 |
|  |  | RS | 43.07±4.76 |  |  |
|  |  | SS | 40.47±5.38 |  |  |
|  | 34 | LS | 68.15±9.37 | 0.661 | 0.718 |
|  |  | RS | 78.15±12.45 |  |  |
|  |  | SS | 62.47±11.12 |  |  |
| SMR | 22 | LS | 39.87±6.09 | 1.587 | 0.452 |
|  |  | RS | 53.09±8.44 |  |  |
|  |  | SS | 39.46±6.89 |  |  |
|  | 28 | LS | 52.66±7.12 | 2.574 | 0.276 |
|  |  | RS | 46.88±5.43 |  |  |
|  |  | SS | 33.86±7.93 |  |  |
|  | 34 | LS | 41.94±7.81 | 0.440 | 0.802 |
|  |  | RS | 43.09±6.16 |  |  |
|  |  | SS | 36.47±6.32 |  |  |
| MMR | 22 | LS | 27.65±3.88 | 0.429 | 0.807 |
|  |  | RS | 27.28±4.29 |  |  |
|  |  | SS | 31.61±4.58 |  |  |
|  | 28 | LS | 41.95±5.96 | 0.672 | 0.715 |
|  |  | RS | 33.77±4.75 |  |  |
|  |  | SS | 41.01±6.33 |  |  |
|  | 34 | LS | 27.67±5.55 | 0.870 | 0.647 |
|  |  | RS | 33.32±5.36 |  |  |
|  |  | SS | 34.78±4.52 |  |  |
| AAS | 22 | LS | 42.43±7.09 | 0.625 | 0.731 |
|  |  | RS | 35.68±4.98 |  |  |
|  |  | SS | 43.43±8.41 |  |  |
|  | 28 | LS | 48.43±5.50 | 0.988 | 0.610 |
|  |  | RS | 40.39±5.81 |  |  |
|  |  | SS | 53.58±9.06 |  |  |
|  | 34 | LS | 45.77±7.35 | 0.677 | 0.713 |
|  |  | RS | 42.43±8.97 |  |  |
|  |  | SS | 53.07±6.76 |  |  |
| No. of Feeds | 22 | LS | 124.07±38.39 | 2.272 | 0.321 |
|  |  | RS | 135.55±49.20 |  |  |
|  |  | SS | 48.99±35.36 |  |  |
|  | 28 | LS | 79.37±13.51 | 0.222 | 0.895 |
|  |  | RS | 78.74±11.10 |  |  |
|  |  | SS | 67.09±10.53 |  |  |
|  | 34 | LS | 102.97±21.35 | 0.184 | 0.912 |
|  |  | RS | 82.61±14.52 |  |  |
|  |  | SS | 101.06±25.93 |  |  |
| Feeding latency | 22 | LS | 53.97±19.70 | 0.099 | 0.951 |
|  |  | RS | 60.58±21.42 |  |  |
|  |  | SS | 48.99±19.19 |  |  |
|  | 28 | LS | 97.33±18.81 | 0.635 | 0.728 |
|  |  | RS | 81.19±12.14 |  |  |
|  |  | SS | 114.30±16.21 |  |  |
|  | 34 | LS | 100.13±20.24 | 0.622 | 0.732 |
|  |  | RS | 115.86±23.37 |  |  |
|  |  | SS | 75.25±17.69 |  |  |
| Activity | 22 | LS | 47.65±8.96 | 1.265 | 0.531 |
|  |  | RS | 42.52±5.42 |  |  |
|  |  | SS | 57.19±10.14 |  |  |
|  | 28 | LS | 49.55±6.86 | 2.767 | 0.251 |
|  |  | RS | 83.82±10.82 |  |  |
|  |  | SS | 71.81±9.78 |  |  |
|  | 34 | LS | 38.18±7.11 | 3.089 | 0.213 |
|  |  | RS | 45.73±6.47 |  |  |
|  |  | SS | 64.58±10.42 |  |  |
| Exploration | 22 | LS | 52.51±6.55 | 0.874 | 0.646 |
|  |  | RS | 66.15±7.71 |  |  |
|  |  | SS | 52.88±7.68 |  |  |
|  | 28 | LS | 32.49±6.05 | 5.266 | 0.072 |
|  |  | RS | 62.75±12.01 |  |  |
|  |  | SS | 51.85±7.58 |  |  |
|  | 34 | LS | 27.85±4.75 | 3.439 | 0.179 |
|  |  | RS | 47.69±4.90 |  |  |
|  |  | SS | 43.84±6.89 |  |  |
| Boldness | 22 | LS | 123.22±18.64 | 1.417 | 0.565 |
|  |  | RS | 113.33±15.43 |  |  |
|  |  | SS | 186.23±34.84 |  |  |
|  | 28 | LS | 157.15±26.29 | 1.182 | 0.554 |
|  |  | RS | 122.55±19.59 |  |  |
|  |  | SS | 238.33±56,77 |  |  |
|  | 34 | LS | 98.25±18.71 | 1.485 | 0.476 |
|  |  | RS | 136.33±36.97 |  |  |
|  |  | SS | 194±38.73 |  |  |
| Fecundity | 22 | LS | 63.98±25.19 | 0.101 | 0.951 |
|  |  | RS | 81.22±17.91 |  |  |
|  |  | SS | 95.33±47.66 |  |  |
|  | 28 | LS | 69.64±16.68 | 2.940 | 0.230 |
|  |  | RS | 19.89±7.35 |  |  |
|  |  | SS | 63.04±14.53 |  |  |
| Egg size | 22 | LS | 4.29±0.47 | 4.054 | 0.131 |
|  |  | RS | 5.62±0.43 |  |  |
|  |  | SS | 4.71±0.54 |  |  |
|  | 28 | LS | 4.32±0.29 | 6.691 | **0.035*** |
|  |  | RS | 5.60±0.58 |  |  |
|  |  | SS | 4.13±0.30 |  |  |
| Hatch day | 22 | LS | 30.55±2.45 | 13.977 | **0.0009***** |
|  |  | RS | 21.13±2.28 |  |  |
|  |  | SS | 14.71±1.76 |  |  |
|  | 28 | LS | 25.26±1.60 | 0.029 | 0.985 |
|  |  | RS | 25.76±3.56 |  |  |
|  |  | SS | 25.57±2.18 |  |  |
| Larvae size | 22 | LS | 8.83±1.15 | 0.954 | 0.620 |
|  |  | RS | 8.63±0.70 |  |  |
|  |  | SS | 7.45±0.87 |  |  |
|  | 28 | LS | 9.81±0.64 | 0.188 | 0.910 |
|  |  | RS | 10.27±1.41 |  |  |
|  |  | SS | 10.24±1.09 |  |  |
| CTmax | 22 | LS | 2.05±0.31 | 4.723 | 0.094 |
|  |  | RS | 3.03±0.46 |  |  |
|  |  | SS | 1.55±0.27 |  |  |
|  | 28 | LS | 2.45±0.39 | 0.349 | 0.836 |
|  |  | RS | 2.10±0.32 |  |  |
|  |  | SS | 2.49±0.49 |  |  |
|  | 34 | LS | 6.36±0.92 | 0.481 | **0.008**** |
|  |  | RS | 7.53±1.21 |  |  |
|  |  | SS | 4.93±0.68 |  |  |
